# The study of etiological and demographic characteristics of neonatal mortality and morbidity - a consecutive case series study from Pakistan

**DOI:** 10.1186/1471-2431-12-131

**Published:** 2012-08-27

**Authors:** Nabeel Manzar, Bushra Manzar, Anum Yaqoob, Muneer Ahmed, Jai Kumar

**Affiliations:** 1 Department of Pediatrics, Dow University of Health Sciences, Karachi, Pakistan; 2 B-3, Paradise Apartments, Sarwar Shaheed Road, Karachi, Pakistan

**Keywords:** Neonatal, Mortality, Morbidity, Causes, Demographic factors, Bacteriological spectrum

## Abstract

**Background:**

To determine the etiology, management, bacteriological spectrum and outcome of neonatal patients admitted in Civil Hospital Karachi (CHK) and to examine the factors associated with it.

**Methods:**

This hospital based descriptive study of 1463 patients from both sexes who were admitted to Paediatric department, CHK from 1st January 2008 till 31st December 2010 with an established cause according to modified Wigglesworth classification and fulfilling other inclusion criteria were included in the study. Data regarding their demographic profile and potential risk factors was collected on a well structured proforma. Cases were followed until discharge or expiry. Data was analyzed using descriptive statistics.

**Results:**

The male to female ratio in our study was 1.12:1. Seven hundred and thirty-four patients were delivered at home (50.2%) and 1010 were less than 7 days old (69%). Out of the total cohort of expired subjects, 89 participants (74.8%) were < 7 days of life. Mortality was more in neonates born at home in rural areas to illiterate mother; 74 patients (62.2%). Most of the deaths; 57 were in neonates suffering from specific infections (47.9%) followed by 38 deaths in immaturity group (31.9%) and 19 related to asphyxial conditions (15.9%). The most common isolates were Staphylococcus aureus (28.7%) followed by Klebsiella (24.8%) and Pseudomonas aeruginosa (16.6 ). One hundred and nineteen (8.13%) of the neonates died in our study group.

**Conclusions:**

These results suggest that neonates with illiterate mothers with high parity and below average socioeconomic level were more susceptible to mortality in the early neonatal period. Most of the cases of mortality were due to specific infections.

## Background

Every year 4 million neonates die during the first four weeks of life [1]. Out of these mortalities, 99% take place in the developing countries of the world, where there are a lack of proper health care facilities [2]. Pakistan accounts for 7% of the global neonatal mortality with an estimated 298000 neonatal deaths annually and a reported mortality rate of 56 per 1000 live births [3]. Infection, immaturity and asphyxia account for 87% of neonatal deaths worldwide [4]. Bacterial sepsis is considered to be an important cause of neonatal morbidity and mortality. The organisms isolated in the developed part of the world in cases of sepsis differ greatly from those seen in developing countries and sometimes within the same countries the organism isolated are very different. With the ever changing knowledge of bacteriological spectrum, simple and inexpensive interventions are the need of the time during prenatal, natal and postnatal period to counter these organisms. These interventions include proper nutrition, immunization and supplementation of the pregnant mother followed by skilled delivery, early breast feeding of the neonate and in case of morbidity, appropriate management of the neonate to prevent mortality [5-8]. One of the United Nation’s Millennium Development Goal aspires to reduce the under 5 childhood mortality to 30 per 1000 live births by 2015 and since 41% of all deaths in children under 5 years of age is shared by neonatal deaths, our focus should be on reducing neonatal mortality [9]. In order to achieve this goal we need to address all the factors associated with neonatal morbidity and mortality. New evidence suggests that demographic factors like maternal education, socioeconomic and parity status also plays an important role in neonatal mortality and morbidity [10]. A lot of studies on neonatal morbidity and mortality are available from the developed countries, however, there is a paucity of data from developing countries like Pakistan because there is neither a national database nor any relevant authority to collect and standardize the data but individual studies have been carried out in local cities in the past. These studies have many limitations and have mainly focused on neonatal sepsis while ignoring other important factors contributing to high neonatal mortality and morbidity rate. The magnitude of the problem coupled with the parents anguish as well as cost of admission and the treatment expenditure incurred to the state compelled us to carry out our research. Factors such as patient’s age, gender, weight, presenting symptoms, treatment and their outcome in terms of mortality and morbidity as well as demographic factors were taken into consideration. The main objective of our study was to determine the etiology, management, bacteriological spectrum and outcome of neonatal patients admitted in Civil Hospital, Karachi (CHK) and to examine the factors associated with it.

## Methods

This hospital based case series study was carried at Pediatrics Department, CHK, Pakistan from 1st January 2008 till 31st December 2010. CHK is a tertiary care hospital and caters to patients coming from both urban and rural parts of Sindh. A total of 1463 neonatal patients diagnosed with different etiologies and who fulfilled the inclusion criteria were included in the study. The tudy protocol was reviewed and approved by the ethics committee at the study centre (Dow University of Health Sciences-Ethical Review Committee) and the study was carried out in accordance with the declaration of Helsinki of 1975, revised in 1983. Patients fulfilling the following study criteria were enrolled in the study: 1) Subjects brought alive to emergency room of pediatric department. 2) Patients of aged up to 1 month with an established diagnosis. 3) Participants of all gender as determined by filled Proforma with a definite history of symptomatology and demographic information. 4) Informed consent from family for participating in the study. Patients lost to follow-up, referred, left against medical advice, brought dead or antepartum fetal deaths were excluded from the study. The study was carried out in two parts, first all patients had their detailed medical history taken with complete physical examination followed by data collection by the investigators on a well structured proforma regarding their social and demographic characteristics. Any complication that occurred during stay in hospital was also recorded. Cases were followed until discharge or expiry of patient. Review of microbiology laboratory database for all blood samples was carried out. All blood samples were transported to the microbiology laboratory at University Hospital without delay for microscopy, culture, and sensitivity testing according to the laboratory’s standard operative procedure. Neonatal period was defined as a period from birth upto 28 days of life for at term babies and up to 44 weeks of gestational age in preterm babies [11]. Gestational age was confirmed from the mother by the last date of menstrual period (LMP) or obstetrical ultrasonography (US). In cases where there was a mismatch in dates, the gestational age by US was considered final. Classification of subjects was done according to age, sex, weight, place of birth, etiology, presentation, potential risk factors, socioeconomic status [12] and outcome criteria. With regards to the causative agent modified Wigglesworth hierarchical classification was used to get a single cause [13]. These factors in hierarchical order are: genetic, maternal, pregnancy related, obstetric or infant related. Case definitions have also been adapted from Wigglesworth and NICE studies [13,14] and the order of the subgroups is strictly hierarchical except for stillbirth’s which have been excluded from this study. Subjects were further divided into two age groups (1) ≤7 days in age (2) >7 days. Weight at presentation was divided into five groups (1) Extremely Low Birth Weight (<0.75 kg) (2) Very Low Birth Weight (<1.5 kg) (3) Low Birth Weight (<2.5 kg) (4) Normal (≥2.5 kg) (5) Large for gestational age (>4 kg). The subject’s place of birth was also classified into home based delivery or hospital based. The neonatal morbidity and mortality rate were defined as the proportion of neonates brought sick and expired during the study period respectively. The potential demographic and risk factors were also classified accordingly (Table 1). Data were entered in Statistical Package for Social Sciences (SPSS version 16). Analysis of the data was done using descriptive statistics. Descriptive statistics were computed according to the type of the variable. The means (standard deviations) was computed for continuous variables, while categorical variables were assessed by computing frequencies. 

**Table 1:** Demographic and risk factors in the household of the subjects

**Variable**	**Number (1463)**	**Frequency (%)**
**Maternal age (years)**		
<20	229	15.6
20–35	1078	73.7
>35	156	10.7
**Maternal Education**		
Illiterate	1154	78.8
High School	277	18.9
Graduate	32	2.3
**Socioeconomic status**		
Below Average	1186	81.1
Average	249	17.0
Above Average	28	1.9
**Parity**		
1	438	29.9
2–5	805	55.1
>6	220	15.0
**Living Area**		
Urban	1137	77.7
Rural	326	22.3

## Results

A total of 1463 subjects based on the inclusion criteria were incorporated in the study. One hundred and thirty three patients were excluded. Twenty eight (21.1%) patients left against medical advice, 36 (27.1%) were referred, 16 (12%) were brought dead while 53 (39.8%) were lost to follow-up. Out of the total subjects, there were 776 (53%) males and 687 (47%) females aged up to 28 days (mean ± std.dev = 5.12 ± 1.3 days). The male to female ratio was almost 1.12:1 with a mean weight of 2.42 +/− 1.5 kg. A total of 734 (50.2%) patients admitted were those delivered at home and 1010 (69%) neonates were less than 7 days old. Four hundred and ninety-five (33.8%) neonates weighed more than 2.5 kg followed by 424 (28.9%) low birth neonates, 247 (16.8%) very low birth weight infants and 209 (14.4%) extremely low birth weight subjects. The mortality rate was 8.13% in the current study. The baseline characteristics of subjects are presented in Table 2. Out of the total cohort of expired subjects, 89 (74.8%) participants were < 7 days of life in age while 30 (25.2%) patients were more than 7 days old. The overall mortality was more in those born at home (62.2%) than compared to those given birth at hospital (37.8%). Neonates with extremely low birth weight had greater morbidity and mortality (41.2%) as compared to normal (11.7%), LBW (18.5%) and VLBW (26.1%) neonates. Most of the neonates admitted were with some specific condition (53.9%) followed by asphyxia related condition (22.3%) and immaturity related conditions (22%) while others were either malformation (1.2%) or unclassifiable (0.6%). Table 3 shows the causative factors responsible for admission and mortality in neonates as per modified Wigglesworth hierarchical classification. Highest numbers of deaths; 57 (47.9%) were seen in the specific condition group. Most of these were infection related to sepsis (28.6%), pneumonia (4.9%), meningitis (4.1%) and gastroenteritis (2.7%). This was followed by 38 (31.9%) deaths in Immaturity group and 19 (15.9%) deaths in asphyxia group. Positive cultures were obtained in 205 (14.01%) of the patients, despite many patients being on antibiotic therapy prior to culture. Whereas, 213 (14.6%) subjects had clinical suspicion of sepsis but were culture negative. Gram-negative bacteria were the most common group of bacteria isolated. The most common isolate was Staphylococcus aureus (28.7%). Other organisms isolated included Streptococcus pneumonia (8.5%), Klebsiella pneumonia (24.8%), Pseudomona aeruginosa (16.6%), Escherichia coli (11.2%), Enterobacter (8.3%) and candida albanicus (1.9%). The micro-organisms cultured from haemotological samples are presented in Table 4. The choice of antibiotics was guided by the antibiotic policy of the Paediatric unit. For the majority of the patients, a third-generation cephalosporin (cefotaxime or ceftriaxone) plus an aminoglycoside (amikacin) was started empirically. The outcome of the patients was determined in terms of either discharged home or death. 1344 (91.9%) patients were discharged satisfactorily. Majority of the neonates admitted had illiterate mothers (78.8%), belonged to a below average household (81.1%) and most of them lived in the urban area (77.7%). Further mortality pattern was mostly the same as the morbidity pattern except that the neonates of rural area 72 (60.5%) were more susceptible to death.

**Table 2:** Baseline characteristics of patients

**Variable**	**No. of patients**	**Frequency**	**Mortality**	**Frequency**
	**(n = 1463)**	**(%)**	**(n = 119)**	**(%)**
**Age (months)**				
≤7 days	1010	69	89	74.8
>7 days	453	31	30	25.2
Mean ± std. dev	5.12 ± 1.3 days			
**Sex**				
Male	776	53	63	52.9
Female	687	47	56	47.1
**Place of birth**				
Home	734	50.2	74	62.2
Hospital	729	49.8	45	37.8
**Weight**				
Normal	495	33.8	14	11.7
ELBW	209	14.4	49	41.2
VLBW	247	16.8	31	26.1
LBW	424	28.9	22	18.5
LGA	88	6.1	03	2.5
Mean ± std. dev	2.42 ± 1.5 kg			
**Outcome**				
Discharged	1344	91.9		
Expired	119	8.1		
**Timing of neonatal death**				
Early (0–7 days)	94	78.9		
Late (8–28 days)	25	21.1		

**Table 3:** Neonatal morbidity and mortality based on modified Wigglesworth’s hierarchical classification

**Causative Factor**	**No. of patients**	**Frequency (%)**	**Mortality**	**Frequency (%)**
	**(1463)**		**(119)**	
**Lethal Malformation**	17	1.2	03	2.5
**Specific condition**	789	53.9	57	47.9
**Asphyxia related**	326	22.3	19	15.9
**Immaturity**	322	22	38	31.9
**Unclassifiable**	09	0.6	02	1.8

**Table 4:** Culture positive bacterial isolates from patients

**Organism**	**No. of Patients**	**Frequency**
	**N = 205**	**(%)**
**Gram Positive Bacteria**		
Streptococcus pneumonia	**17**	**8.5**
Staphylococcus aureus	**59**	**28.7**
**Gram Negative Bacteria**		
Pseudomona aeruginosa	**34**	**16.6**
Enterobacter	**17**	**8.3**
Klebsiella	**51**	**24.8**
E. coli	**23**	**11.2**
**Fungal**	**04**	**1.9**

## Discussion

The neonatal period carries the highest risk of death in human life [4]. The risk of children dying under the age of five has fallen, but the number of deaths in neonatal period has actually increased [10]. In the present study, neonates with illiterate mothers with high parity and below average socioeconomic level were more susceptible to mortality in the early neonatal period. This is due to the fact that in low income countries there is a major focus on maternal death and under 5 children deaths but less attention has been paid to neonatal morbidity and mortality [15]. Neonatal deaths represent an increasing proportion of under 5 deaths [16]. Male to female ratio in our study was similar to other such studies carried all over the world [17,18]. The majority of neonates admitted were less than 7 days of life which is comparable to a study done in France in which the mean age of neonates was 7 ± 1 day [19]. A study carried out by Afsheen et al. documented a morbidity rate of 82.1% in neonates less than 7 days old and early neonatal mortality was also high as has been seen in this study [18]. The mortality rate in our study was 8.13% which is comparatively low when compared to 13% [20], 15% [19], 16.4% [9], and 9.6% [18], from studies carried out in Nigeria, France, India and Pakistan respectively. However, this figure must be taken with caution since some of the patients were referred or left against medical advice and hence the actual mortality rate may be higher than seen in the study. In our study, majority of the patients that died were those that were home delivered (62.2%) in rural areas potentially because of the delay in reaching the center as well as the unhygienic cord practices and lack of proper antenatal care [21]. A study carried out in squatter settlements of Karachi showed a 43.9% rate of birth at homes [18]. However, in our study the rate of delivery at home is more. This may be due to the fact that study center also serves a large part of rural Sindh. These traditional birth practices are still very common in rural parts of Sindh, where local midwives are not properly trained to handle birth care leading to delayed presentation and high mortality. In our study, highest mortality was seen in neonates with specific conditions (infections). Similar findings have been observed in Africa where 38.3% of morbidity and 43.7% of mortality was attributed to neonatal infection and sepsis [21]. Eighteen percent, 16.8% and 91% of deaths in Nigeria, France and Northern parts of India have been attributed to specific infections respectively [19,20,22]. Furthermore, our finding that infections including sepsis, pneumonia and meningitis, are important contributors to neonatal deaths is consistent with recent studies from developing countries and emphasizes the importance of monitoring delivery and hospital acquired infections [23]. Immature neonates contributed the second most common cause of neonatal death which was consistent with other studies in which prematurity and LBW were the major factors [20,24]. In a study carried out in rural India, preterm deliveries contributed to 30% and infection based deaths contributed to 25% of deaths taking place in the early neonatal period. However the proportion of deaths due to infection based causes tend to increase in the late neonatal period [10]. In another study carried out in Lahore most of the deaths were attributed to prematurity (11.4%) followed by asphyxia (7%) and infection (4.2%) [25]. Majority of the deaths due to preterm delivery can be prevented by proper antenatal care and by promoting maternal health [26]. Antenatal care four times during pregnancy by a skilled medical provider is recommended by WHO since 1944 [27]. and is shown to be associated with improved neonatal morbidity and mortality [28,29]. Policy and programme attention is shifting towards a maternal, newborn, and child health (MNCH) continuum of care, instead of competing calls for mother or child, the focus is on universal coverage of effective interventions, integrating care throughout the lifecycle and building a comprehensive and responsive health system.

Gram negative infections (62.9%) were more common than gram positive organisms (37.1). The most common gram positive isolate was Staphylococcus aureus (28.7%) while Klebsiella was the most common isolate (22.9%) in the gram negative group. A study carried out on neonatal sepsis in Pakistan documented S. aureus to be the most common organism in the gram positive group however E. coli was the commonest agent seen in the gram negative group [30]. Similarly, in another study conducted by Rabia et al. Enterobacter was the commonest gram negative isolate while S. aureus was seen as the commonest gram positive isolate [17]. This shows the variable spectrum of bacteriological isolates seen in various studies and even in studies in different cities of the same country. Evidence has shown that certain demographic and social factors are implicated in neonatal mortality and morbidity. In this study, neonates with illiterate multiparous mothers of below average socioeconomic status were prone to mortality and morbidity. This conclusion has also been drawn in other studies carried out in South Asia [10,18].

Finally this study fulfills the objective set by the study protocol for this project of assessing the socio-demographic factors and causative agents of neonatal morbidity and mortality as well as the bacteriological spectrum and outcome of subjects brought to CHK. This study holds important implications for public health and highlights the high prevalence of morbidity and mortality in the Pakistani neonate population. However there remain certain limitations due to the hospital based nature of the study as well as the mortality rate was potentially less conclusive due to the exclusion of patients lost to follow-up, brought dead, referred or left against medical advice. In addition CHK receives patients from other cities and rural areas, so this may not represent the true statistics of the area of our study as well as cannot be generalized for the whole population. A large scale prospective multi-center study with appropriate power is recommended for further evaluating the ethnicity, geographic differences and other risk factors for neonatal mortality and morbidity in underdeveloped countries like Pakistan.

## Conclusion

On the basis of our study we conclude that the most common cause of neonatal mortality was due to specific infections. Gram negative bacteria were the most common organisms isolated. Early neonatal period was the time when neonates were most susceptible to a high mortality rate. Literacy rate of mothers correlated significantly with neonatal morbidity with the highest 78.8% of cases in neonates with illiterate mothers. In most of the cases neonates belonged to rural mothers with high parity and below average socioeconomic level.

## Competing interests

All authors declare that they have no competing of interest.

## Financial disclosure

None to declare.

## Authors’ contribution

NM, JK and MA conceived the study, participated in its design and coordination. NM and AY performed the data collection and statistical analysis. NM, BM and AY drafted the manuscript. BM participated in the design of the study. All the authors read and approved the final manuscript.

## Pre-publication history

The pre-publication history for this paper can be accessed here:

http://www.biomedcentral.com/1471-2431/12/131/prepub
